# Silicone ring tourniquet could be a substitute for a conventional tourniquet in total knee arthroplasty with a longer surgical field: a prospective comparative study in simultaneous total knee arthroplasty

**DOI:** 10.1186/s12891-023-06469-9

**Published:** 2023-05-09

**Authors:** Tae sung Lee, Kwan Kyu Park, Byung Woo Cho, Woo-Suk Lee, Hyuck Min Kwon

**Affiliations:** 1 Department of Orthopedic Surgery, Severance Hospital, Yonsei University College of Medicine, 50-1, Yonsei-ro, Seodaemun-gu, Seoul, 03722 Korea; 2 Department of Orthopedic Surgery, Gangnam Severance Hospital, Yonsei University College of Medicine, Seoul, Korea

**Keywords:** Total knee arthroplasty, Pneumatic tourniquet, Silicon ring tourniquet

## Abstract

**Introduction:**

This study aimed to compare the clinical outcomes of silicon ring tourniquets and conventional pneumatic tourniquets in total knee arthroplasty (TKA). The study compared the operation time, total bleeding amount, length from the tourniquet distal end to the patella superior pole (L_TP), and complications related to the two tourniquet application methods and attempted to determine whether the silicon ring tourniquet has advantages over conventional pneumatic tourniquets.

**Materials and methods:**

This prospective comparative study included 30 patients who underwent bilateral simultaneous TKA for degenerative osteoarthritis in August to December 2021. All patients underwent TKA on one side with a conventional pneumatic tourniquet, while TKA on the other side with a silicon ring tourniquet. The primary outcomes were the L_TP, operation time, tourniquet time, total bleeding amount, total drainage amount, and postoperative visual analog scale (VAS) score of the tourniquet applied site at 6, 24, and 48 h postoperatively. The secondary outcome was tourniquet-related complications in both groups.

**Results:**

L_TP was significantly longer in the silicon ring tourniquet group compared with that in the pneumatic tourniquet group (20.22 ± 2.74 cm versus 15.12 ± 2.40, p < 0.001). No significant difference was found in other results. The tourniquet applied site pain was less in the silicon ring tourniquet group (p = 0.037).

**Conclusions:**

Silicon ring tourniquet application resulted in better clinical outcomes than conventional pneumatic tourniquets in TKA. Because we can obtain a wider surgical field using silicon ring tourniquets without complications, silicon ring tourniquets could be a substitute for conventional pneumatic tourniquets in total knee arthroplasty or distal femoral surgeries.

## Introduction

In total knee arthroplasty (TKA), a pneumatic tourniquet is commonly used to reduce surgical time and intraoperative blood loss to create a clearer surgical field, increasing cement penetration for stronger cement fixation [1–6]. However, some studies have shown that postoperative complications, such as deep vein thrombosis (DVT), ischemic pain, nerve palsy, and surgical site infection rates, are relatively higher in the tourniquet group [3, 7]. Despite the advantages of tourniquet use, its effectiveness remains meaningful, and orthopedic surgeons usually use tourniquets in lower extremity surgery [8, 9]. There are many types of tourniquets (conventional pneumatic tourniquet, sterile silicon ring tourniquet, rubber tourniquet, sterile pneumatic tourniquet). Conventional pneumatic tourniquets are economical because they are reusable, easily deflated, and reflated during the operation. However, owing to the uneven pressure of the pneumatic tourniquet, thigh pain or local skin complications may occur when a tourniquet is applied after surgery [10–12]. Moreover, a pneumatic tourniquet is about 106 mm long and is sufficient to restrict the operation field in the proximal femur. The average femur length of Caucasian women is 413 ± 22 mm, whereas that of Asian women is 380 ± 18 mm. The average femur length of Caucasian men is 446 ± 22 mm, whereas that of Asian men is 418 ± 20 mm [13]. This means that the conventional pneumatic tourniquet length about 100 mm (106 mm) can hinder the surgical field of the proximal thigh, especially in revisional TKA or distal femur fracture surgery, especially in Asian patients: some surgeons omit the tourniquet for better visualization of the surgical field in distal femur fractures where the fracture line extends to the mid-shaft of the femur.

Silicon ring tourniquets are developed to overcome the disadvantages of conventional pneumatic tourniquets [14, 15]. A silicon ring tourniquet consists of a silicon ring wrapped within an elastic sleeve and two straps, and it is applied in the operative field under sterile conditions, unlike pneumatic tourniquets. The silicon ring tourniquet is short, which results in a longer operative field in the proximal thigh area [16]. It is not only small but can also obtain accurate and even pressure; thus, there will be fewer complications in the clinical use of silicon ring tourniquets [17, 18]. Moreover, the pressure of the tourniquet is 350 mmHg on average and can be easily controlled by removing it in the operation field.

This study aimed to compare the clinical outcomes of silicon ring tourniquets and conventional pneumatic tourniquets in TKA. We compared the operation time, total bleeding amount, length from the tourniquet distal end to the patella superior pole (L_TP), and complications related to tourniquet application of the two tourniquet application methods and attempted to determine whether the silicon ring tourniquet has advantages over conventional pneumatic tourniquets.

## Materials and methods

### Data collection

**Fig. 1:**
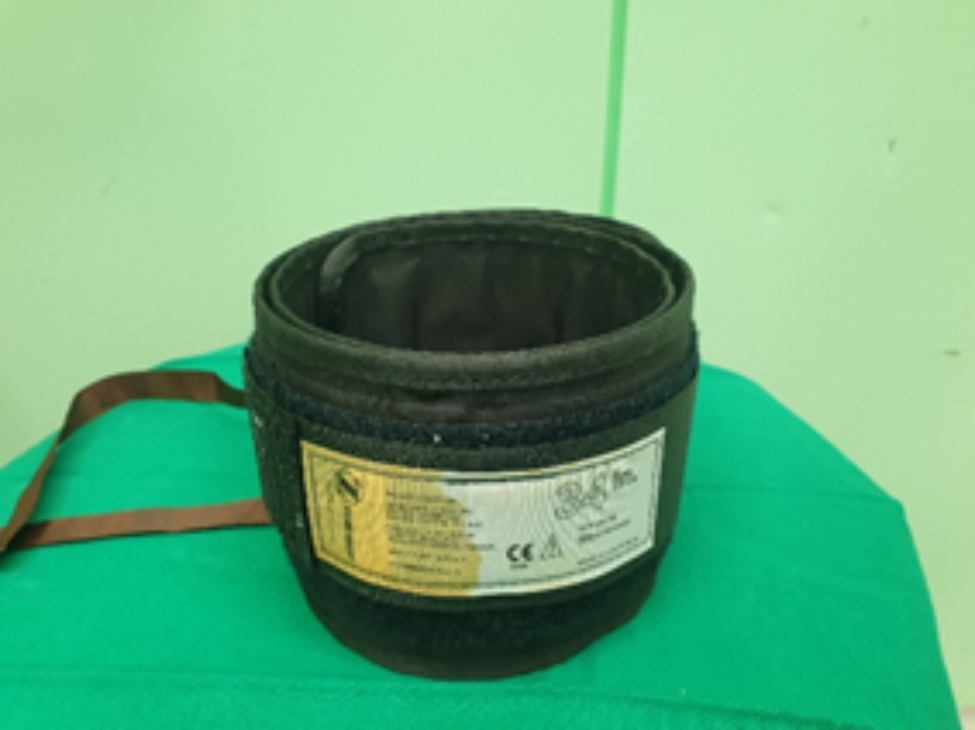
Zimmer A.T.S.®/Zimmer Biomet, America

**Fig. 2:**
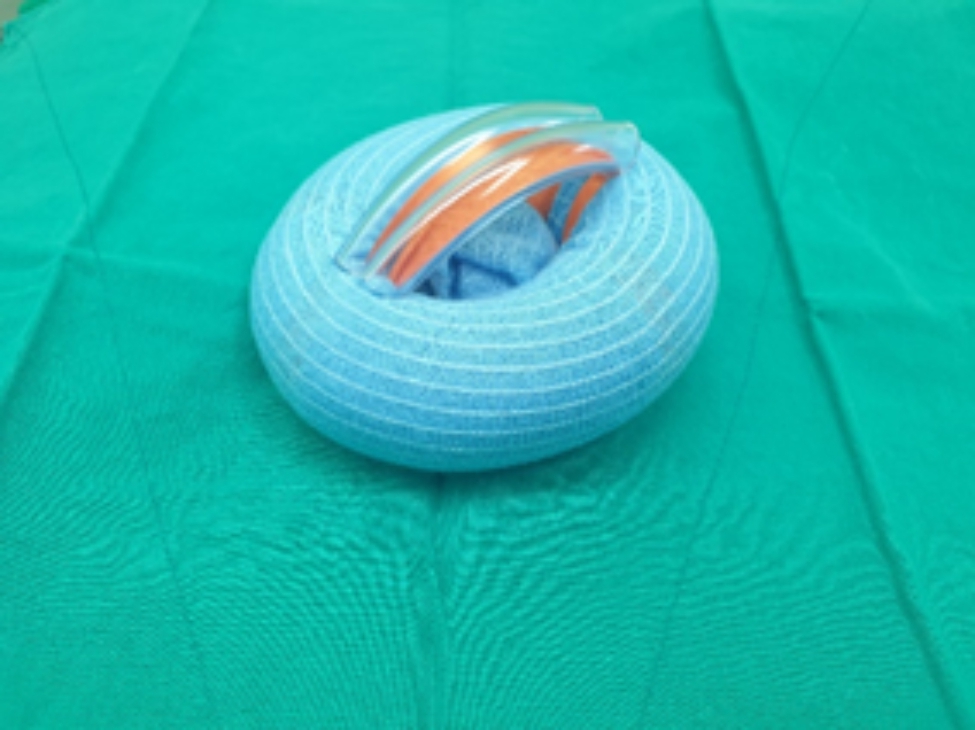
Rapband ®/RapMedicare, Korea

**Fig. 3:**
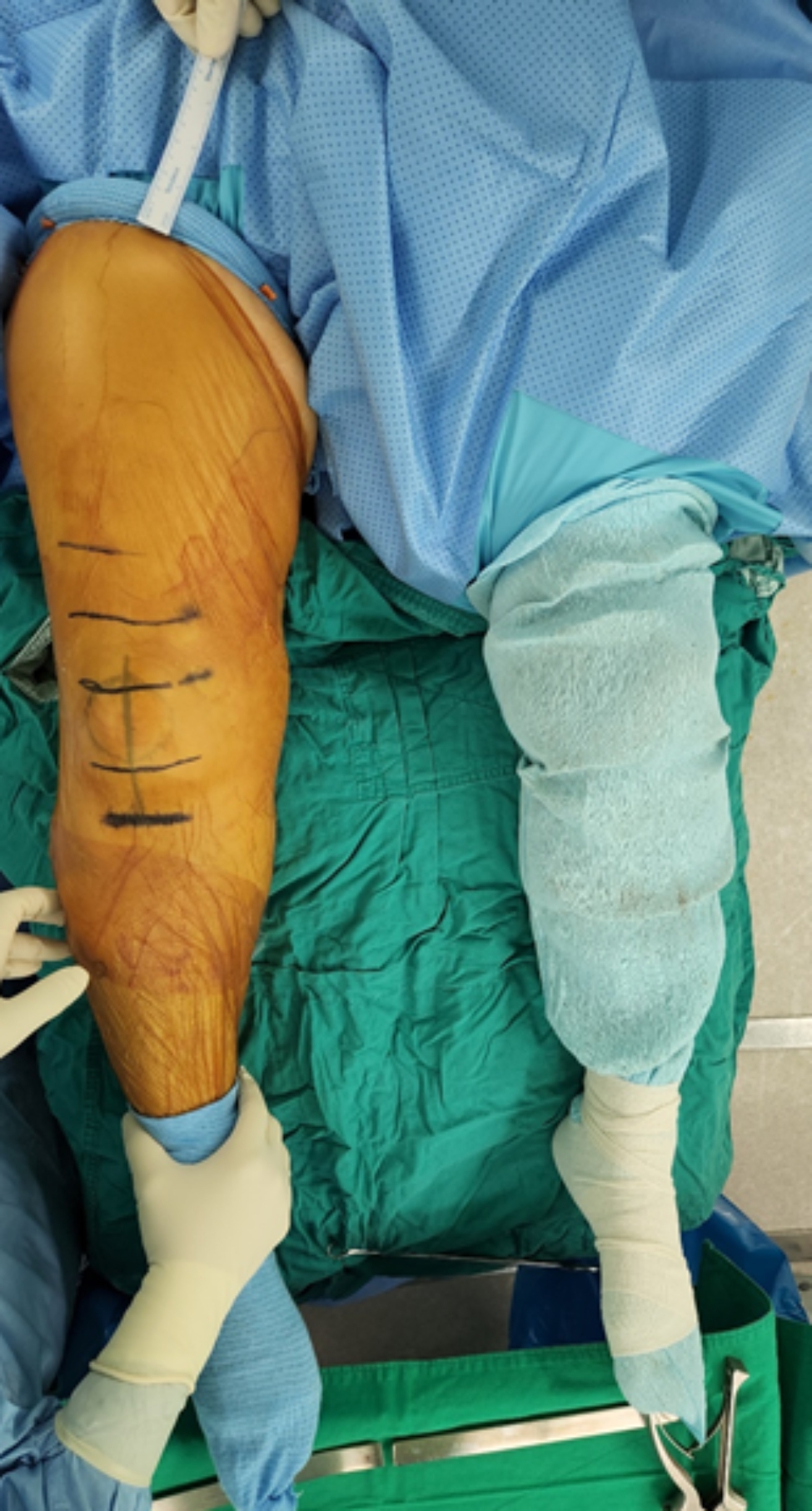
Intraoperative comparison of silicon ring tourniquet (right) and pneumatic tourniquet (left) in bilateral simultaneous total knee arthroplasty

After Institutional Review Board approval was obtained, 30 patients who underwent bilateral simultaneous TKA in 2021 were prospectively included, and their electronic medical records were collected at a single tertiary hospital. Patients not suited for tourniquet application due to allergy to the tourniquet or other contraindications were excluded. In all patients, one lower extremity received a conventional pneumatic tourniquet (Zimmer A.T.S.®/Zimmer Biomet, America) with a standard 86 × 10 cm and a standard pressure of 320 mmHg (Fig. 1), whereas the other side of the lower extremity had a silicon ring tourniquet (Rapband ®/RapMedicare, Korea) (Figs. 2 and 3). All silicon ring tourniquets were size XL (pressure directed by the manufacturer, 320 ± 20 mmHg for size XL). We used size XL tourniquets for all patients to ensure that the applied pressure was as similar as possible to the conventional pneumatic tourniquet with a standard pressure of 320 mmHg. Patients for whom the lower extremity was applied a silicon ring tourniquet were randomly assigned using an Excel program. Patients with inflammatory knee arthritis, including rheumatoid arthritis, knee joint infection, revision surgery, severe instability, anatomical deformity, or bone defects, were excluded.

All patients received standardized general or spinal anesthesia. Thirty minutes before the end of the surgery, IV fentanyl (1 µg/kg) and palonosetron (0.075 mg) were administered to the patient for postoperative analgesia and antiemetic effects, respectively. IV PCA that comprised 7 µg/kg of fentanyl and 0.075 g of palonosetron (total volume including saline: 100 mL) was administered for 48 h postoperatively in all patients and was delivered as a 2 mL/h background infusion and in 0.5 mL doses upon patient demand with a 15-minute lockout time. In the ward, all patients received celecoxib (200 mg) orally followed by acetaminophen (1 g) intravenously every 12 h. All patients in both groups received the same pain management regimen postoperatively. [19]

### Outcome measurements

Demographic data, L_TP, operation time, tourniquet time, total bleeding amount, total drainage amount, and postoperative visual analog scale (VAS) score at the tourniquet applied site in each leg at 6, 24, and 48 h after surgery were assessed in all patients. The postoperative thigh pain and local skin complications in the tourniquet application area were compared. Osteoarthritis grade, Kellgren and Lawrence (KL) grade, preoperative hip–knee–ankle (HKA) angle on radiography, preoperative length of the mechanical axis of the femur on radiography (L_fMA), and circumference of the tourniquet-applied area in the upper thigh were assessed in all patients.

### Statistical analysis

Chi-square and t-tests were performed to compare the two tourniquet application methods. To obtain a power of 0.95 (1-β) with an α of 0.05, the calculated sample size was 27 cases per group [20, 21]. Considering a dropout rate of 10%, the target sample size was 30 cases per group. McNemar’s test was performed for analysis. Statistical analyses were performed using IBM SPSS Statistics for Windows, Version 25.0 (IBM Corp., Armonk, NY, USA), and p-values of < 0.05 were considered significant.

## Results

The demographic data are shown in Table 1. The baseline characteristics of the patients were identical because each patient’s lower extremity and the other were compared. The age of the patients was a mean of 69.2 ± 4.9 years. Almost all patients were women; only 3 patients were men because osteoarthritis of the knee is predominant among women. Patient BMI was a mean of 27.7 ± 3.8 (Table 1). No significant difference was found in the KL grade, HKA angle, L_fMA, and circumference of the tourniquet-applied area in the upper thigh (Table 2). Kellgren–Lawrence grade was 3 or 4: 4 patients were grade 3, and 26 patients were grade 4 in the silicon ring tourniquet group. Meanwhile, 6 patients were grade 3, and 24 patients were grade 4 in the pneumatic tourniquet group. One patient had osteoarthritis of different stages in each of their knees. Hip-knee-ankle angle showed a mean of 9.20 ± 5.41 in the silicon ring tourniquet group and 9.88 ± 5.78 in the pneumatic tourniquet group. The length of mechanical axis of the femur was a mean of 411.05 ± 25.60 in the silicon ring tourniquet group and 409.04 ± 24.14 in the pneumatic tourniquet group. The circumferences of the upper thigh were almost identical between the two groups.

**Table 1:** Patients’ demographics

Parameters	
Numbers of patients (knees)	30 (60)
Age (years)	69.2 ± 4.9 (60–80)
Male patients (%)	3 (10%)
BMI (kg/m^2^)	27.7 ± 3.8 (20.4–37)
ASA grade	
1	3 (10%)
2	14 (46.7%)
3	13 (43.3%)

**Table 2:** Preoperative evaluation of silicon ring tourniquet group and conventional pneumatic tourniquet group

	Silicon ring tourniquet	Pneumatic tourniquet	p
Kellgren - Lawrence grade			0.317
3	4 (13.3%)	6 (20%)	
4	26 (86.6%)	24 (80%)	
Hip-knee-ankle angle (°)	9.20 ± 5.41	9.88 ± 5.78	0.485
The length of mechanical axis of femur (cm)	411.05 ± 25.60	409.04 ± 24.14	0.200
Circumference of upper thigh (cm)	52.8 ± 4.32	52.6 ± 4.16	0.326

The L_TP was significantly longer in the silicon ring tourniquet-applied lower extremity than in the pneumatic tourniquet-applied lower extremity (20.22 ± 2.74 cm versus 15.12 ± 2.40, p < 0.001) (Table 3). The ratio of exposed operative field to the entire thigh length was a mean of 49.2 ± 6.1% in the silicon ring tourniquet and 36.9 ± 5.6% in the pneumatic tourniquet, the difference of which was significant. However, no significant differences were noted in the operation time, tourniquet time, total bleeding amount, total drainage amount, and VAS of the tourniquet applies site at 6, 24, and 48 h after surgery (Table 3).

**Table 3:** Comparison of clinical outcomes between silicon ring tourniquet group and conventional pneumatic tourniquet group

	Silicon ring tourniquet	Pneumatic tourniquet	p
The length from tourniquet distal end to patella superior pole (cm)	20.22 ± 2.74	15.12 ± 2.40	< 0.001
Ratio of exposed operative field to entire thigh length (%)	49.2 ± 6.1	36.9 ± 5.6	< 0.001
Operation time (minutes)	52.60 ± 8.20	51.10 ± 5.58	0.230
Tourniquet time (minutes)	55.33 ± 6.15	54.07 ± 3.96	0.325
Total bleeding amount (ml)	47.3 ± 15.00	44.10 ± 18.16	0.200
Total drainage amount (ml)	476.61 ± 203.55	436.59 ± 191.83	0.242
Visual analog scale (VAS)			
At postoperative 6 h	0.87 ± 0.82	0.97 ± 1.03	0.326
At postoperative 24 h	0.77 ± 0.82	0.90 ± 1.03	0.211
At postoperative 48 h	0.27 ± 0.58	0.30 ± 0.60	0.662

**Fig. 4:**
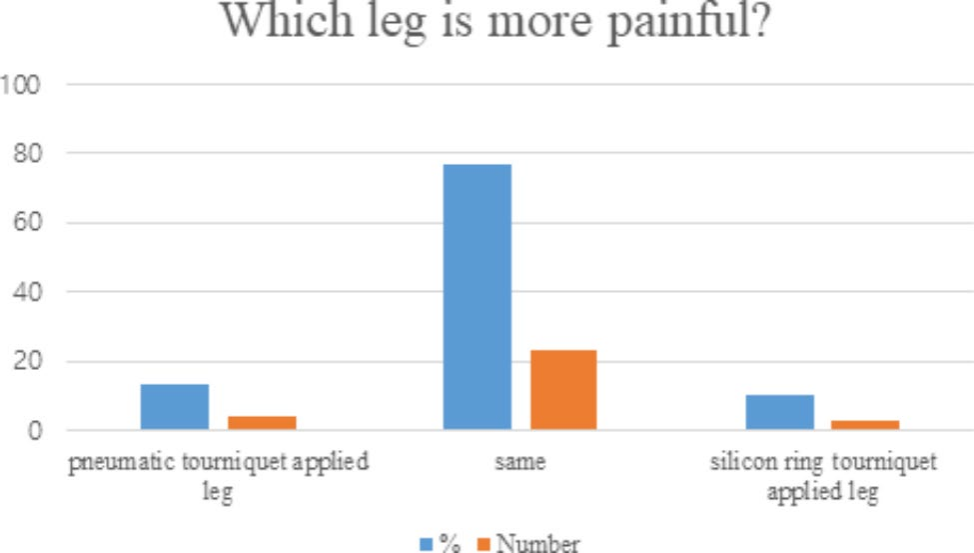
Patient’s reply of which leg’s pain is higher

As regards the patient’s report for which leg pain was higher, 4/30 (13.3%) patients felt higher pain in the pneumatic tourniquet-applied lower extremity, 23/30(76.7%) patients felt the same, and 3/30(10%) patients experienced higher pain in the silicon ring tourniquet-applied lower extremity (Fig. 4). No local complications related to the tourniquet (blistering or local skin complications, nerve complications) were observed in either group, and the thigh pain in the area where the tourniquet was applied was less in the silicon ring tourniquet (p = 0.037) (Table 4).

**Table 4:** Local complication related to tourniquet between silicon ring tourniquet group and conventional pneumatic tourniquet group

	Silicon ring tourniquet	Pneumatic tourniquet	p
Thigh pain in tourniquet area	4 (13.3%)	11 (36.7%)	0.037
Blistering or local skin complication	1 (3.3%)	3 (10%)	0.301
Nerve complication	0 (0%)	1 (3.3%)	0.313
Other complications (Postoperative infection, Vessel complications)	0 (0%)	0 (0%)	1.000

## Discussion

### Main findings

The most important finding of this study was that the length from the distal tourniquet end to the patella superior pole was significantly longer in the silicon ring tourniquet group than in the pneumatic tourniquet group. In addition, in the same patients, the thigh pain when the silicon ring tourniquet was applied was less than the thigh pain when the conventional pneumatic tourniquet was applied. Although no difference was noted between the two tourniquets in terms of clinical results related to pain or postoperative bleeding, this study showed that the silicon ring tourniquet had advantages in a wider surgical field and thigh pain-related tourniquet application without complications than conventional pneumatic tourniquets.

### Previous studies

Silicon ring tourniquets are widely used in other fields of surgery owing to their advantages [17]. Drogos et al. [22] introduced a new tourniquet device, the silicon ring tourniquet, which is effective in orthopedic surgery. Jenny et al. [10] showed a decreased rate of skin complications with silicon ring tourniquet despite no significant change in the calculated blood loss. Sanjay et al. [23] showed that the advantages of the silicone ring tourniquet include less local pain, no local skin problems, and accurate tourniquet pressure at the application site. Of 50 patients in whom the conventional tourniquet was applied, 8 showed local bruising, and 2 had blister formation, resulting in a local skin site complication rate of 20%. All previous studies have only focused on clinical outcomes of silicon ring tourniquet use.

### Effectiveness of a silicon ring tourniquet

Although only few studies have compared the conventional pneumatic tourniquet with the silicon ring tourniquet, no studies have focused on the exact length of the surgical field from the tourniquet and preoperative features of the lower extremity. This was a single-center, prospective study that focused on the effectiveness of a silicon ring tourniquet in lower extremity surgery. The operation time, total blood loss, total drainage amount, and postoperative VAS of the tourniquet applies site showed no significant differences in this study. However, L_TP showed a significant difference (51 mm longer in the silicon ring tourniquet). In primary TKA, a longer operative field does not influence the surgical field. However, in cases of revision or complex TKA with stiffness or distal femur fracture surgery, a longer operative field is very important that some surgeons give up tourniquets to make longer surgical drapes. The difference of 51 mm is 13.42% of the femur length in Asian women and 12.20% in Asian men, which can influence a better surgical field. In this situation, silicon ring tourniquets can substitute conventional pneumatic tourniquets. In addition, in a pneumatic tourniquet, pressure is applied on a two-line applied site and then diffused, which can be very painful at the pressure-applied site [24]. However, the pressure by silicon ring tourniquet is evenly applied around 360°, which can be less painful to the patients [18]. It can explain this study’s result, silicon ring tourniquet showed lesser thigh pain in tourniquet applied site. Although postoperative pain score was not significantly different, less local pain could help patients for faster recovery after surgery. In a previous study, [25] intraoperative knee range of motion measurement can be underestimated when TKA surgery is performed; therefore, longer exposure of the thigh during TKA could more accurately check the intraoperative knee range of motion, especially in revisional TKA or stiffness of knee cases. Park et al. [26] described that the use of a silicone ring tourniquet in minimally invasive plate fixation for distal femoral fractures decreased the amount of intraoperative bleeding, compared to no use of a tourniquet. Further research ought to focus on distal femur fracture cases or other lower extremity surgeries.

The strength of this study is prospective comparative study. We compared results of both tourniquet methods in one patient, and randomization of which leg is applied by silicon ring tourniquet, which could reduce the patient dependent bias. Using statistics, we proved that L_TP difference and the ratio of exposed operative field to entire thigh length were significantly larger with the silicon ring tourniquet. And thigh pain on tourniquet applied site was significantly lower in silicon ring tourniquet.

### Limitations

This study had some limitations. First, VAS scores in simultaneous TKA can affect each other in one patient because pain is a complex mechanism. However, randomization of the tourniquet type in the left or right leg can hide the patient’s bias. Second, the pressure on the silicon ring tourniquet can differ depending on the circumference of the proximal thigh. Although we compared the circumference of the proximal thigh where the tourniquet was applied and found no significant difference, the larger the circumference of the proximal thigh, the greater the pressure on the lower extremity, which can influence postoperative pain. Finally, the ideal L_TP difference between the pneumatic tourniquet (106 mm) and the silicon ring tourniquet (25 mm) was 81 mm, but the result (51 mm) was shorter than expected. If we applied a silicon ring tourniquet more proximally, the difference would be much longer.

## Conclusions

Silicon ring tourniquet application resulted in better clinical outcomes than conventional pneumatic tourniquets in TKA. Because we can obtain a wider surgical field using silicon ring tourniquets without complications, silicon ring tourniquets could be a substitute for conventional pneumatic tourniquets in total knee arthroplasty or distal femoral surgeries.

## Data Availability

The datasets used and/or analyzed during the current study are available from the corresponding author upon reasonable request.

## References

[1] Cai DF, Fan QH, Zhong HH, Peng S, Song H (2019). The effects of tourniquet use on blood loss in primary total knee arthroplasty for patients with osteoarthritis: a meta-analysis. J Orthop Surg Res.

[2] Hegde V, Bracey DN, Johnson RM, Dennis DA, Jennings JM (2021). Tourniquet Use improves cement penetration and reduces Radiolucent Line Progression at 5 years after total knee arthroplasty. J Arthroplasty.

[3] Yi S, Tan J, Chen C, Chen H, Huang W (2014). The use of pneumatic tourniquet in total knee arthroplasty: a meta-analysis. Arch Orthop Trauma Surg.

[4] Zhang W, Li N, Chen S, Tan Y, Al-Aidaros M, Chen L (2014). The effects of a tourniquet used in total knee arthroplasty: a meta-analysis. J Orthop Surg Res.

[5] Tai TW, Chang CW, Lai KA, Lin CJ, Yang CY (2012). Effects of tourniquet use on blood loss and soft-tissue damage in total knee arthroplasty: a randomized controlled trial. J Bone Joint Surg Am.

[6] Dennis DA, Kittelson AJ, Yang CC, Miner TM, Kim RH, Stevens-Lapsley JE (2016). Does Tourniquet Use in TKA affect recovery of lower extremity strength and function? A Randomized Trial. Clin Orthop Relat Res.

[7] Watanabe H, Kikkawa I, Madoiwa S, Sekiya H, Hayasaka S, Sakata Y (2014). Changes in blood coagulation-fibrinolysis markers by pneumatic tourniquet during total knee joint arthroplasty with venous thromboembolism. J Arthroplasty.

[8] Præstegaard M, Beisvåg E, Erichsen JL, Brix M, Viberg B (2019). Tourniquet use in lower limb fracture surgery: a systematic review and meta-analysis. Eur J Orthop Surg Traumatol.

[9] Berry DJ, Bozic KJ (2010). Current practice patterns in primary hip and knee arthroplasty among members of the American Association of hip and knee surgeons. J Arthroplasty.

[10] Jenny JY, Bahlau D, Wisniewski S (2016). Silicone ring tourniquet or pneumatic cuff tourniquet for total knee arthroplasty. Int Orthop.

[11] Kumar N, Yadav C, Singh S, Kumar A, Vaithlingam A, Yadav S (2015). Evaluation of pain in bilateral total knee replacement with and without tourniquet; a prospective randomized control trial. J Clin Orthop Trauma.

[12] Park JY, Kim SE, Lee MC, Han HS (2020). Elastic pneumatic tourniquet cuff can reduce postoperative thigh pain after total knee arthroplasty: a prospective randomized trial. BMC Musculoskelet Disord.

[13] Thiesen DM, Ntalos D, Korthaus A, Petersik A, Frosch KH, Hartel MJ. A comparison between Asians and Caucasians in the dimensions of the femoral isthmus based on a 3D-CT analysis of 1189 adult femurs. Eur J Trauma Emerg Surg 2021. 10.1007/s00068-021-01740-x PMC9192442 34319407

[14] Norman D, Greenfield I, Ghrayeb N, Peled E, Dayan L (2009). Use of a new exsanguination tourniquet in internal fixation of distal radius fractures. Tech Hand Up Extrem Surg.

[15] McEwen JA (1981). Complications of and improvements in pneumatic tourniquets used in surgery. Med Instrum.

[16] Kovar F, Jauregui JJ, Specht SC, Baker E, Bhave A, Herzenberg JE (2016). Upper Extremity nerve function and Pain in human volunteers with narrow versus wide tourniquets. J Long Term Eff Med Implants.

[17] Bourquelot P, Levy BI (2016). Narrow elastic disposable tourniquet (Hemaclear®) vs. traditional wide pneumatic tourniquet for creation or revision of hemodialysis angioaccesses. J Vasc Access.

[18] Lee OS, Lee MC, Han HS (2017). Efficacy and safety of a new elastic tourniquet cuff in total knee arthroplasty: a prospective randomized controlled study. Biomed Eng Online.

[19] Choi YS, Park KK, Lee B, Nam WS, Kim DH. Pericapsular Nerve Group (PENG) Block versus Supra-Inguinal Fascia Iliaca Compartment Block for Total Hip Arthroplasty: A Randomized Clinical Trial. J Pers Med 2022, 12(3). 10.3390/jpm12030408 PMC8951338 35330408

[20] Faul F, Erdfelder E, Buchner A, Lang AG (2009). Statistical power analyses using G*Power 3.1: tests for correlation and regression analyses. Behav Res Methods.

[21] Faul F, Erdfelder E, Lang AG, Buchner A (2007). G*Power 3: a flexible statistical power analysis program for the social, behavioral, and biomedical sciences. Behav Res Methods.

[22] Drosos GI, Ververidis A, Mavropoulos R, Vastardis G, Tsioros KI, Kazakos K (2013). The silicone ring tourniquet in orthopaedic operations of the extremities. Surg Technol Int.

[23] Bhalchandra Londhe S, Vinod Shah R, Sanjay Londhe S, Agrawal PO, Antao NA, Churhe S (2021). Comparison of local pain and tissue reaction between conventional pneumatic tourniquet and disposable silicone ring tourniquet during total knee arthroplasty. J Clin Orthop Trauma.

[24] Estebe JP, Le Naoures A, Chemaly L, Ecoffey C (2000). Tourniquet pain in a volunteer study: effect of changes in cuff width and pressure. Anaesthesia.

[25] Kwon HM, Yang IH, Lee WS, Yu ARL, Oh SY, Park KK (2019). Reliability of intraoperative knee range of motion measurements by Goniometer compared with Robot-Assisted arthroplasty. J Knee Surg.

[26] Park K-B, Jin H-K, Hwang I-Y, Chang S-W, Na S-C (2020). Does the use of a silicone Ring Tourniquet Help reduce bleeding in the minimally invasive internal fixation with locking plate for distal femoral fractures?. J Korean Fract Soc.

